# Reduced Endothelial Leptin Signaling Increases Vascular Adrenergic Reactivity in a Mouse Model of Congenital Generalized Lipodystrophy

**DOI:** 10.3390/ijms221910596

**Published:** 2021-09-30

**Authors:** Thiago Bruder-Nascimento, Taylor C. Kress, Matthew Pearson, Weiqin Chen, Simone Kennard, Eric J. Belin de Chantemèle

**Affiliations:** 1 Vascular Biology Center, Medical College of Georgia at Augusta University, Augusta, GA 30912, USA; BRUDER@pitt.edu (T.B.-N.); tkress@augusta.edu (T.C.K.); MPEARSON@augusta.edu (M.P.); skennard@augusta.edu (S.K.); 2 Department of Pediatrics, Division of Endocrinology, Center for Pediatric Research in Obesity and Metabolism (CPROM), University of Pittsburgh, Pittsburgh, PA 15260, USA; 3 Vascular Medicine Institute (VMI), University of Pittsburgh, Pittsburgh, PA 15260, USA; 4 Department of Physiology, Medical College of Georgia at Augusta University, Augusta, GA 30912, USA; WECHEN@augusta.edu; 5 Department of Medicine, Division of Cardiology, Medical College of Georgia at Augusta University, Augusta, GA 30912, USA

**Keywords:** lipodystrophy, adipose tissue, leptin, vascular function

## Abstract

The adipokine leptin, which is best-known for its role in the control of metabolic function, is also a master regulator of cardiovascular function. While leptin has been approved for the treatment of metabolic disorders in patients with congenital generalized lipodystrophy (CGL), the effects of chronic leptin deficiency and the treatment on vascular contractility remain unknown. Herein, we investigated the effects of leptin deficiency and treatment (0.3 mg/day/7 days) on aortic contractility in male Berardinelli-Seip 2 gene deficient mice (gBscl2^-/-^, model of CGL) and their wild-type control (gBscl2^+/+^), as well as in mice with selective deficiency in endothelial leptin receptor (LepR^EC-/-^). Lipodystrophy selectively increased vascular adrenergic contractility via NO-independent mechanisms and induced hypertrophic vascular remodeling. Leptin treatment and Nox1 inhibition blunted adrenergic hypercontractility in gBscl2^-/-^ mice, however, leptin failed to rescue vascular media thickness. Selective deficiency in endothelial leptin receptor did not alter baseline adrenergic contractility but abolished leptin-mediated reduction in adrenergic contractility, supporting the contribution of endothelium-dependent mechanisms. These data reveal a new direct role for endothelial leptin receptors in the control of vascular contractility and homeostasis, and present leptin as a safe therapy for the treatment of vascular disease in CGL.

## 1. Introduction

Lipodystrophy is a heterogeneous metabolic disease of congenital or acquired origin characterized by a total or partial absence of adipose tissue [1,2,3,4] at the origin of major metabolic derangements including severe insulin resistance, type 2 diabetes, hypertriglyceridemia, and steatohepatitis [2,4,5,6]. Lipodystrophy is not only a metabolic disease, but also a major risk factor for cardiovascular disorders. The prevalence of hypertension, cardiac hypertrophy, left ventricle dysfunction, aortic valve calcification, and heart failure is high in patients with any form of lipodystrophy [2,7,8,9,10,11,12,13,14]. However, the etiopathology of cardiovascular disorders remain ill-defined in patients with lipodystrophy.

One of the hallmarks of lipodystrophy is a marked decrease in adipose mass. As the adipose tissue is the primary source of the hormone leptin, lipodystrophy is characterized by a striking reduction in circulating leptin levels [4,5,15]. Leptin has initially been identified as a key regulator of metabolic function. Leptin regulates glucose tolerance, insulin sensitivity, and ultimately body weight via central and peripheral mechanisms involving activation of the sympathetic nervous system, and control of food intake and energy expenditure [4,5,11,15,16,17]. Leptin receptors are ubiquitously expressed and its involvement extends beyond the metabolic system, especially to the reproductive [18], immune [19], bone [20] and cardiovascular system [11,15,21,22,23,24,25,26]. Leptin is notably a key regulator of vascular function [15,21,22,27]. Recent work by our group showed that selective reduction in endothelial leptin signaling reduces nitric oxide (NO) bioavailability and contributes to vascular inflammation, whereas selective increases in endothelial leptin signaling and leptin supplementation fully restore endothelial function via direct regulation of vascular redox signaling [15,21,28,29]. However, whether reduced leptin levels contribute to alteration in vascular contractility remains unknown. The goal of the present study is to analyze the effects of lipodystrophy and leptin deficiency on vascular contractility in the *Bscl2* deficient mouse, a unique mouse model of congenital generalized lipodystrophy (CGL).

## 2. Results

### 2.1. Lipodystrophy Specifically Increases Vascular Adrenergic Contractility

**Figure 1:**
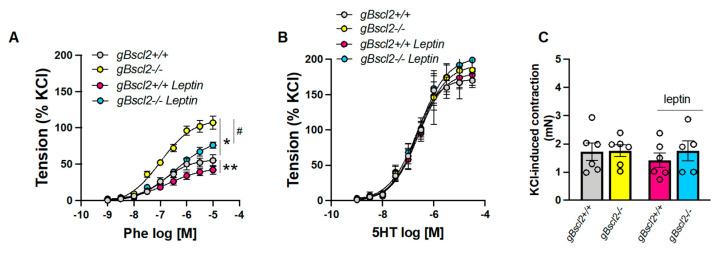
Leptin treatment reduces vascular adrenergic contractility in aortas from lipodystrophic mice. Concentration response curves to phenylephrine (**A**), serotonin (**B**) and KCl (**C**) in aortic rings from wild type (gBscl2*^+/+^*) or Bscl2 deficient mice (gBscl2*^-/-^*) treated or not with leptin (0.3 mg/kg/day for 1 week, via osmotic mini-pump). Data are presented as mean ± S.E.M. N = 4–7; * *p* < 0.05 gBscl2*^+/+^* vs. gBscl2*^-/-^*; ** *p* < 0.05 gBscl2*^+/+^* vs. gBscl2*^+/+^* + leptin; # *p* < 0.05 gBscl2*^-/-^* vs. gBscl2*^-/-^* + leptin.

To determine the effects of lipodystrophy on vascular contractility, aortic rings were submitted to KCl and concentration response curves to phenylephrine (Phe) and serotonin (5HT). As reported in Figure 1, lipodystrophy markedly increased Phe-induced constriction in aortas from gBscl2*^-/-^* mice, but did not alter vascular responses to either KCl or 5HT. Previous work by our group demonstrated that reduced leptin levels and endothelial leptin signaling lead to endothelial dysfunction in gBscl2*^-/-^* mice, which is reversed by leptin supplementation [15,21,28,29]. Therefore, we investigated the effects of restoring leptin levels via leptin infusion on vascular contractility. Remarkably, leptin treatment significantly reduced Phe-mediated constriction in both gBscl2*^-/-^* and gBscl2*^+/+^* mice and almost restored vascular Phe-contractility to baseline in gBscl2*^-/-^* mice (Figure 1A). However, chronic leptin infusion was without effects on KCl- and 5HT-induced constriction (Figure 1B,C).

### 2.2. Lipodystrophy Increases Arterial Media Thickness and Collagen Deposition 

**Figure 2:**
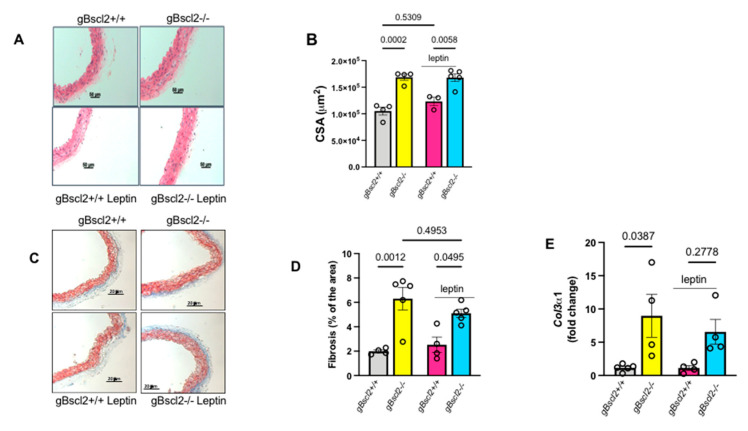
Leptin treatment does not restore vascular remodeling and fibrosis in lipodystrophic mice. Hematoxylin and Eosin staining (**A**), cross-sectional area quantification (**B**), Masson’s trichrome staining (**C**), fibrosis quantification (**D**), and collagen 3α1 (Col3α1) gene expression (**E**) in thoracic aortas from wild-type (gBscl2*^+/+^*) or Bscl2 deficient mice (gBscl2*^-/-^*) treated or not with leptin (0.3 mg/Kg/day for 1 week, via osmotic mini-pump). Data are presented as mean ± S.E.M. N = 3–5. *p* < 0.05 is considered statically different between groups.

To investigate the potential origin of increased vascular adrenergic contractility, we obtained indices of vascular remodeling. As reported in Figure 2A,B, lipodystrophy is associated with an increase in arterial cross-sectional area (CSA) which remains elevated under chronic leptin treatment in gBscl2*^-/-^* mice. Similarly, lipodystrophy is associated with increased vascular fibrosis as illustrated by elevations in Masson’s trichrome staining (Figure 2C,D) and measurements of collagen content (Figure 2E). Leptin treatment did not alter vascular fibrosis or Col3α1 level, indicating that leptin supplementation reduces vascular contractility independent of changes in vascular structure.

### 2.3. Lipodystrophy Increases Vascular Adrenergic Contractility via Nox1-Dependent Mechanisms

**Figure 3:**
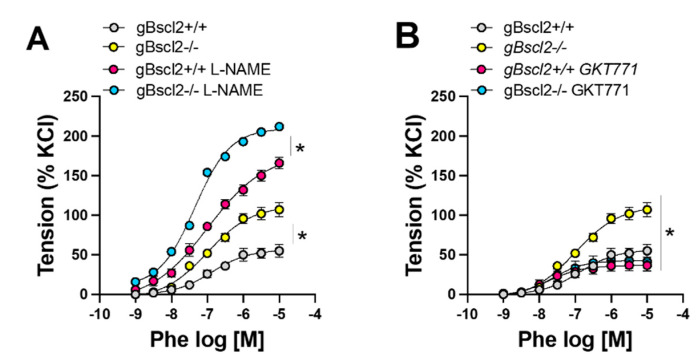
Lipodystrophy-associated increases in vascular adrenergic contractility are eNOS-independent but Nox1-mediated. Concentration response curves to phenylephrine in aortic rings from wild-type (gBscl2*^+/+^*) or Bscl2 deficient mice (gBscl2*^-/-^*) in the presence of N-nitro-l-arginine methyl ester [L-NAME; (100 μmol/L)] (**A**) or GKT771 (10 μmol/L)] (**B**). Data are presented as mean ± S.E.M. N = 3–6. * *p* < 0.05 vs. gBscl2*^-/-^*. *p* < 0.05 gBscl2*^+/+^* vs. gBscl2*^-/-^* in presence of L-NAME.

We, and others, have shown that leptin has the capacity to increase NO bioavailability in endothelial cells by activating NO synthase (NOS) or reducing NADPH oxidase 1-derived reactive oxygen species (ROS) production [15,22]. Therefore, to investigate the potential contribution of the endothelium and ROS in the increase in Phe-mediated constriction, we repeated the concentration response to Phe in the presence of either L-NAME or the selective Nox1 inhibitor GKT771 [15,30]. L-NAME significantly increased Phe-mediated vasoconstriction in both gBscl2*^+/+^* and gBscl2*^-/-^*, but preserved the difference between groups, likely ruling out alterations in NOS activity as a cause of increased adrenergic contractility in gBscl2*^-/-^* mice (Figure 3A). On the other hand, Nox1 inhibition with GKT771 markedly reduced Phe-mediated constriction and restored it to baseline levels (Figure 3B), suggesting that lipodystrophy increases adrenergic contractility via elevated Nox1 activity. 

### 2.4. Leptin Reduces Vascular Adrenergic Contractility via NOS-Dependent Mechanisms in gBscl2^+/+^ Mice

**Figure 4:**
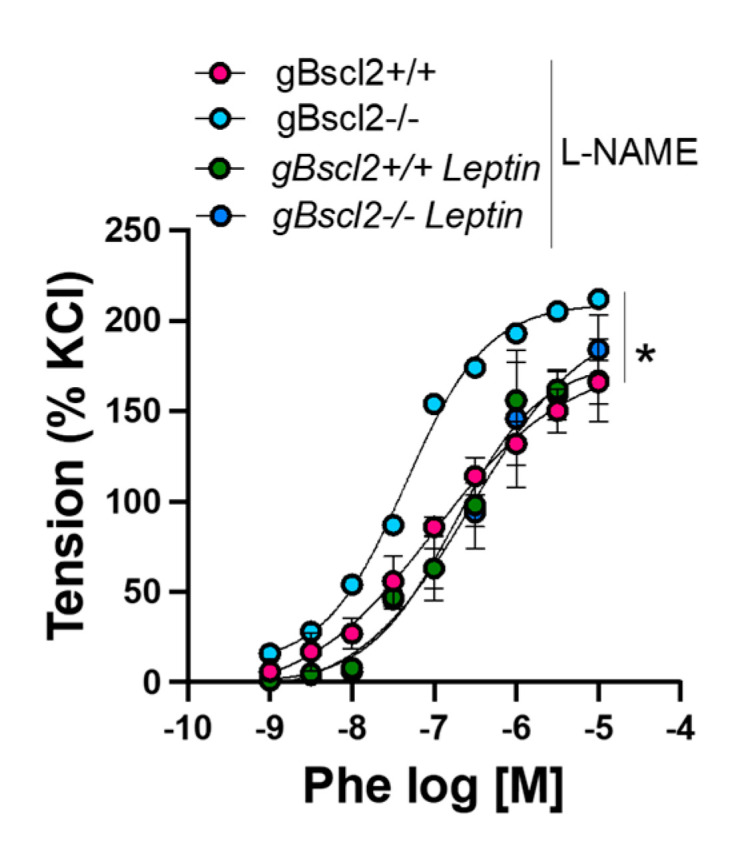
Leptin improves vascular adrenergic contractility in arteries from wild-type mice by regulating nitric oxide bioavailability. Concentration response curves to phenylephrine in aortic rings from wild-type (gBscl2*^+/+^*) and Bscl2 deficient mice (gBscl2*^-/-^*) treated with, or without, leptin (0.3 mg/Kg/day for 1 week, via osmotic mini-pump), and in the presence or absence of N-nitro-l-arginine methyl ester [L-NAME; (100 μmol/L)]. Data are presented as mean ± S.E.M. N = 4–6. * *p* < 0.05 gBscl2*^+/+^* vs. gBscl2*^-/-^* and gBscl2*^-/-^* + L-NAME pre-incubation.

In order to understand the mechanisms whereby leptin reduces Phe-mediated constriction, we repeated the L-NAME curves in aortic rings from leptin treated animals. Remarkably, L-NAME abolished leptin-mediated reduction in Phe-induced constriction in gBscl2*^+/+^* but not in gBscl2*^-/-^* mice (Figure 4), suggesting that leptin treatment reduces vascular adrenergic contractility by increasing NO bioavailability in gBscl2*^+/+^* only, but via different mechanisms in gBscl2*^-/-^* mice. 

### 2.5. Leptin-Mediated Decreases in Adrenergic Contractility Requires Intact Endothelial Leptin Signaling

**Figure 5:**
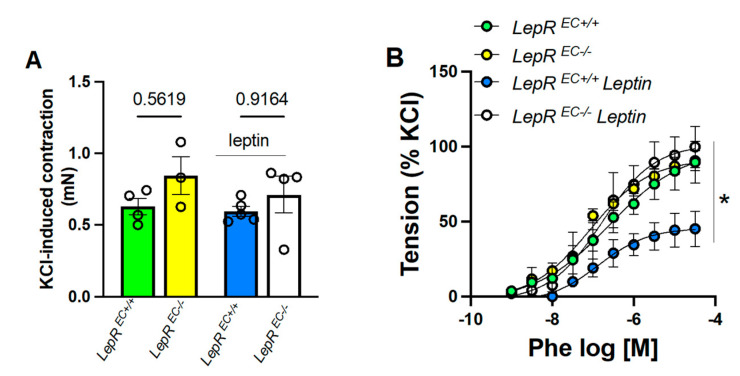
Leptin-mediated reduction in adrenergic vascular contractility requires intact endothelial-leptin signaling. KCl-induced vascular contractility (**A**), and concentration response curves (CRC) to phenylephrine (**B**) in aortic rings from wild type (LepR^EC*+/+*^) and endothelial leptin receptor deficient mice (LepR*^EC-/-^*) treated or not with leptin (0.3 mg/Kg/day for 1 week, via osmotic mini-pump) (**B**). Data are presented as mean ± S.E.M. N = 4. * *p* < 0.05 WT vs. LepR^EC-/-^ mice.

Leptin receptors are ubiquitously expressed [15,16,24]. Therefore, to identify the cell type responsible for the effects of leptin on vascular adrenergic response, we utilized mice deficient in endothelial leptin receptor (LepR^EC-/-^).^.^ These mice showed no alterations in baseline vascular adrenergic contractility or KCL-mediated constriction (Figure 5), but blunted leptin-mediated reduction in Phe-induced constriction, supporting a role for endothelial leptin receptor in leptin-mediated reduction in adrenergic contractility. 

## 3. Discussion

Patients with CGL deficient in adipose tissue and leptin present with severe cardiovascular alterations including hypertension, coronary artery disease, hypertrophic cardiomyopathy and autonomic impairment. Despite numerous studies [2,7,8,9,10,11,12,13,14,31], the consequences of a generalized deficiency in adipose tissue and the subsequent decrease in circulating leptin levels on vascular function remains ill-defined. In the present study, we used a mouse model replicating the human Berardinelli-Seip congenital lipodystrophy syndrome, the Bscl2 deficient mouse [1,15], to characterize their vascular contractile phenotype, and studied the effects of chronic leptin supplementation as patients with CGL would receive [6,11]. Using this approach, we demonstrated that CGL induced a marked increase in vascular adrenergic contractility that is reduced by chronic leptin treatment. We furthered our findings by demonstrating that CGL-mediated increases and leptin-induced reduction in adrenergic contractility are independent of NOS activity, but likely dependent on Nox1. Finally, we showed that the protective effects of leptin treatment require intact endothelial leptin signaling. Relevant to these observations are the mechanisms of impairment of vascular adrenergic reactivity and the mechanisms whereby leptin improves endothelial function. 

Herein, we reported, for the first time, that CGL specifically increases vascular adrenergic contractility. Based on the reported increases in arterial CSA, a global increase in vascular contractility in gBscl2^-/-^ mice could have been expected. However, KCl- and 5HT-mediated constrictions remained intact ruling out morphological changes and vascular remodeling as causes of increased contractility. Similarly, lipodystrophy-associated metabolic alterations, notably hyperglycemia, can likely be excluded as well despite diabetes and hyperglycemia being shown to raise vascular contractility indistinctly of the pathway [32,33]. Therefore, impairment in α-adrenergic signaling within vascular smooth muscle cells (VSMCs) [34,35] or loss of the blunting effects of endothelial cells on α-adrenergic constriction [36,37] may contribute the enhanced vascular adrenergic tone. Using the same animals, we previously investigated the effects of CGL on endothelial function. We initially reported marked impairment in endothelial function caused by a decrease in NO bioavailability. We demonstrated that the reduced NO bioavailability resulted from an increase in Nox1-derived ROS production [15]. Herein, using L-NAME, we ruled out reduced NO bioavailability as a potential contributor to enhanced α_1_-adrenergic contractility but identified increased Nox1 activity as a potential player. Indeed, Nox1 inhibition with the selective Nox1 inhibitor GKT771 [15] fully restored adrenergic contractility in gBscl2^-/-^ mice and abolished the difference between gBscl2^+/+^ and gBscl2^-/-^ mice while L-NAME did not. Further evidence in support of a role for Nox1 is provided by additional results from our group and others demonstrating that Nox1 deficiency in mice protects from increases in adrenergic contractility [21,38]. While these data support the role of Nox1, they do not inform on the cell type responsible for the increase in Nox1 activity. Previous reports indicate that ROS production is required for vascular smooth muscle cell contraction in response to adrenergic receptor stimulation [39]. However, neither Nox1 deficiency [21], nor selective Nox1 inhibition (Figure 3B) blunted α_1_-adrenergic contractility. This indicates that α_1_-adrenergic receptor-mediated ROS production likely derives from other NADPH oxidase isoforms than Nox1 in VSMCs, and potentially suggests the contribution of endothelial cell Nox1. Based on the observation that deficiency in endothelial leptin signaling increases aortic Nox1 expression, as well as the expression of its organizer and activator, NOXO1 and NOXA1 [15], we can speculate that increases in endothelial Nox1 contributes to CGL-associated hyperadrenergic contractility, but further studies are warranted to confirm this hypothesis. 

Herein, we also reported that chronic leptin supplementation restores vascular adrenergic contractility and demonstrated for the first time that leptin-mediated decreases in vascular adrenergic contractility requires intact endothelial leptin signaling. Interestingly, leptin appears to reduce adrenergic contractility via different mechanisms in wild-type and lipodystrophic mice. Consistent with the demonstration that leptin increases vascular NO production [22,27,40], leptin-mediated reduction in adrenergic contractility is abolished by L-NAME supporting its NOS dependency in gBscl2^+/+^ mice. However, L-NAME did not blunt leptin-mediated reduction in adrenergic contractility in gBscl2^-/-^ mice, favoring the contribution of different mechanisms. Nox1 inhibition reduced vascular adrenergic contractility in gBscl2^-/-^ mice (Figure 3B) and leptin decreased vascular Nox1 expression [15,21]. Therefore, the mechanisms may likely be Nox1-dependent. However, while Nox1-mediated increases in adrenergic contractility appear as the most likely explanation for the adrenergic hypercontractility, one cannot rule out vascular adrenergic adaptations in response to changes in vascular sympathetic drive. Indeed, several reports from our group demonstrated that increases in sympathetic tone lead to compensatory decreases in α_1_-adrenergic contractility [41,42], while a reduction in sympathetic tone increases α_1_-adrenergic reactivity [43], notably in response to alteration in leptin levels. As leptin is a major regulator of vascular sympathetic dive [41,42,44] and produced in limited amounts in gBscl2^-/-^ mice [1,15], one can expect that Bscl2 deficient mice exhibit low sympathetic drive and a consequent compensatory increase in vascular adrenergic contractility. Based on the same dogma, we could expect that leptin treatment would reduce vascular adrenergic contractility via raising sympathetic tone. However, addressing the effects of CGL on sympathetic drive was beyond the scope of the present study. 

Increased vascular adrenergic contractility has been reported in humans with type 2 diabetes [45] and aging [46], which are two major risk factors for hypertension, and also in African American individuals [47] who are predisposed to hypertension. Hypertension is currently the leading risk factor for cardiovascular disease [48,49]. Although data in lipodystrophic patients are missing, it is reasonable to speculate that, as observed in mice, lipodystrophic patients exhibits elevated vascular adrenergic tone, which would explain their high propensity to develop hypertension. Interestingly, while leptin treatment reduced vascular adrenergic contractility in mice, it did not reduce blood pressure in lipodystrophic patients [50]. Several factors could explain this discrepancy. First, restoration of metabolic function, notably restoration of euglycemia and improvements in liver function [51,52], which both have negative consequences on blood pressure, could compensate for increases in leptin-mediated sympatho-activation. Second, the present study has been conducted in conductance vessels, which play only minimal roles in blood pressure control. Although we previously demonstrated that leptin similarly regulates vascular function in resistance and conductance arteries [41,42], whether conductance vessels and resistance arteries from lipodystrophic mice respond similarly to leptin remain unknown. Furthermore, leptin supplementation did not improve vascular remodeling (Figure 2), which is crucial for blood flow maintenance via regulating vascular resistance [53,54]. Therefore, we can speculate that the absence of beneficial effects of leptin on arterial stiffness may explain the preservation of the elevated blood pressure in lipodystrophic patients. Finally, blood pressure regulation results from a balance among different variables including a fine tune in renal structure and function [48,49,55]. Liu et al. reported that gBslc2^-/-^ mice present with renal injury [56], which is only restored with large doses of leptin for an extended period of time (1 μg/g/day for 14 days). Indeed, these authors used a dose more than 3 times higher than the dose used in the present study for twice the duration. Then, increasing doses and/or time of leptin treatment could potentially prove to be efficient to decrease blood pressure. Therefore, these results beg for more human and animals studies, in order to better understand the effects of leptin treatment in dose and treatment duration on blood pressure regulation, with a particular focus on the vascular function, and the autonomic control of blood pressure. 

**Figure 6:**
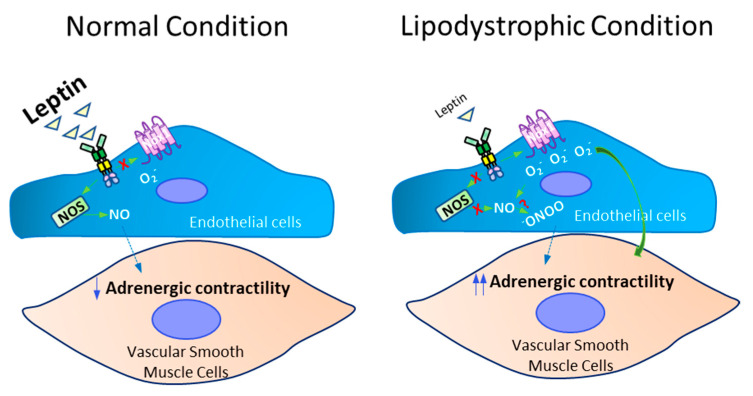
Reduced endothelial leptin signaling increases adrenergic vascular contractility by increasing Nox1-derived ROS. Roles of leptin on vascular adrenergic contractility in physiological and lipodystrophic conditions.

In conclusion, these findings provide the first evidence that reduced leptin levels and endothelial leptin signaling enhanced vascular adrenergic contractility via increases in Nox1-derived ROS (Figure 6). Our data also provide insights into the beneficial effects of leptin supplementation in reducing vascular contractility in an endothelium-dependent manner and identify leptin as a key regulator of vascular tone in physiological conditions. Nevertheless, further studies are necessary to investigate the effects of lipodystrophy and leptin on resistance arteries function, and determine whether higher doses of leptin can reduce blood pressure in patients with lipodystrophy. 

## 4. Materials and Methods

### 4.1. Animals

Mice deficient in Bscl2 (gBscl2^-^^/^^-^) were generated, characterized, and provided by Dr. Weiqin Chen (Department of Physiology, Augusta University). Male animals, 10–12-weeks old, were compared to their wildtype littermate control (gBscl2^+/+^). All animals were fed standard mouse chow, and tap water was provided ad libitum. Mice were housed in an American Association of Laboratory Animal Care-approved animal care facility at Augusta University. Augusta University Institutional Animal Care and Use Committee approved all protocols (IACUC protocol #2011-0108). For tissue collection, mice were anesthetized (isoflurane 5%) and euthanized via decapitation, in accordance with our approved animal protocol.

### 4.2. Leptin Supplementation 

Animals were treated with saline or leptin (0.3 mg/kg/day, ProSpec, Rehovot, Israel) via subcutaneous osmotic mini-pumps (ALZET, Cupertino, Calif; model 1007D, 0.5 μL/h) for 7 days as previously described [15,21,44]. 

### 4.3. Vascular Function

Thoracic aortas were dissected surgically, cleaned of surrounding fat, cut in four rings and mounted on a wire myograph (DMT), as described previously [15,21,42]. Briefly, two tungsten wires were inserted into the lumen of the arteries and fixed to a force transducer and a micrometer. Arteries were bathed in a physiological salt solution and arterial viability was determined with a potassium-rich solution (KCl, 40 mmol/L). Concentration response curves (CRC) to phenylephrine (0.1 nmol/L to 100 μmol/L) and serotonin (5HT, 0.1 nmol/L to 100 μmol/L) were performed in presence or absence of inhibitor N-nitro-l-arginine methyl ester (L-NAME; 100 μmol/L, unspecific nitric oxide synthase (NOS) Sigma Aldrich, MO-USA) or GKT771 (10 μmol/L; specific Nox1 inhibitor, Genkyotex, Saint Julien en Genevois, France). CRCs were normalized by the maximal response evoked by KCl (40 mmol/L). The individual CRC were fitted by nonlinear regression analysis. 

### 4.4. Morphometric Analysis of the Vascular Wall

Thoracic aortas were harvested, cleaned of connective tissue, and fixed in 4% phosphate-buffered paraformaldehyde at pH 7.4 and embedded in paraffin blocks. Four micrometer-thick slices were stained with hematoxylin and eosin (H&E) or Masson’s trichrome stain. Cross-Sectional Area (CSA) was calculated by subtracting the lumen internal area from the external area, which was measured in each tissue section. Fibrosis was quantified by the percentage of fibrotic area in each section. Both parameters were analyzed using ImageJ Program. Stained sections were examined with a light microscopy (ZEISS Axio Imager Observer D1).

### 4.5. Real-Time PCR

Tht total aortic mRNA was extracted (Trizol Plus, Invitrogen, Carlsbad, Calif) and the concentration was established with a NanoDrop 1000 (NanoDrop Technologies, Wilmington, Del). Complementary DNA was generated by RT-PCR with SuperScript III (Thermo Fisher Scientific, NH-USA). Reverse transcription was performed at 50 °C for 50 min; the enzyme was heat inactivated at 85 °C for 5 min, and real-time quantitative RT-PCR was performed with the SYBR-Green Supermix (Bio-Rad Laboratories, Hercules, Calif). Collagen 3α1 (Col3α1, FW: 5′ CCATTTGGAGAATGTTGTGCAAT 3′ and RV: 5′ GGACATGATTCACAGATTCCAGG 3′) and glyceraldehyde 3-phosphate dehydrogenase (GAPDH, FW: 5′ ACCCAGAAGACTGTGGATGG 3′and RV: 5′ CACATTGGGGGTAAGGAACAC 3′.

### 4.6. Statistical Analysis

All data are presented as mean ± SEM. *P* values less than 0.05 were considered significant. Differences in means among groups and treatments, with repeated variables, were compared by 2-way ANOVA with repeated measures, where appropriate. Tukey and Bonferroni tests were used as the post hoc test (GraphPad).

## Data Availability

The data that support the findings of this study are available from the corresponding author on reasonable request.
